# OpenXGR: a web-server update for genomic summary data interpretation

**DOI:** 10.1093/nar/gkad357

**Published:** 2023-05-09

**Authors:** Chaohui Bao, Shan Wang, Lulu Jiang, Zhongcheng Fang, Kexin Zou, James Lin, Saijuan Chen, Hai Fang

**Affiliations:** Shanghai Institute of Hematology, State Key Laboratory of Medical Genomics, National Research Center for Translational Medicine at Shanghai, Ruijin Hospital, Shanghai Jiao Tong University School of Medicine, Shanghai200025, China; Shanghai Institute of Hematology, State Key Laboratory of Medical Genomics, National Research Center for Translational Medicine at Shanghai, Ruijin Hospital, Shanghai Jiao Tong University School of Medicine, Shanghai200025, China; Translational Health Sciences, University of Bristol, BristolBS1 3NY, UK; Bioinformatics Department, School of Life Sciences and Technology, Tongji University, Shanghai200092, China; School of Life Sciences, Central South University, Hunan410083, China; High Performance Computing Center, Shanghai Jiao Tong University, Shanghai200240, China; Shanghai Institute of Hematology, State Key Laboratory of Medical Genomics, National Research Center for Translational Medicine at Shanghai, Ruijin Hospital, Shanghai Jiao Tong University School of Medicine, Shanghai200025, China; Shanghai Institute of Hematology, State Key Laboratory of Medical Genomics, National Research Center for Translational Medicine at Shanghai, Ruijin Hospital, Shanghai Jiao Tong University School of Medicine, Shanghai200025, China

## Abstract

How to effectively convert genomic summary data into downstream knowledge discovery represents a major challenge in human genomics research. To address this challenge, we have developed efficient and effective approaches and tools. Extending our previously established software tools, we here introduce OpenXGR (http://www.openxgr.com), a newly designed web server that offers almost *real-time* enrichment and subnetwork analyses for a user-input list of genes, SNPs or genomic regions. It achieves so through leveraging ontologies, networks, and functional genomic datasets (such as promoter capture Hi-C, e/pQTL and enhancer-gene maps for linking SNPs or genomic regions to candidate genes). Six analysers are provided, each doing specific interpretations tailored to genomic summary data at various levels. Three enrichment analysers are designed to identify ontology terms enriched for input genes, as well as genes linked from input SNPs or genomic regions. Three subnetwork analysers allow users to identify gene subnetworks from input gene-, SNP- or genomic region-level summary data. With a step-by-step user manual, OpenXGR provides a user-friendly and all-in-one platform for interpreting summary data on the human genome, enabling more integrated and effective knowledge discovery.

## Graphical Abstract

**Graphical Abstract:**
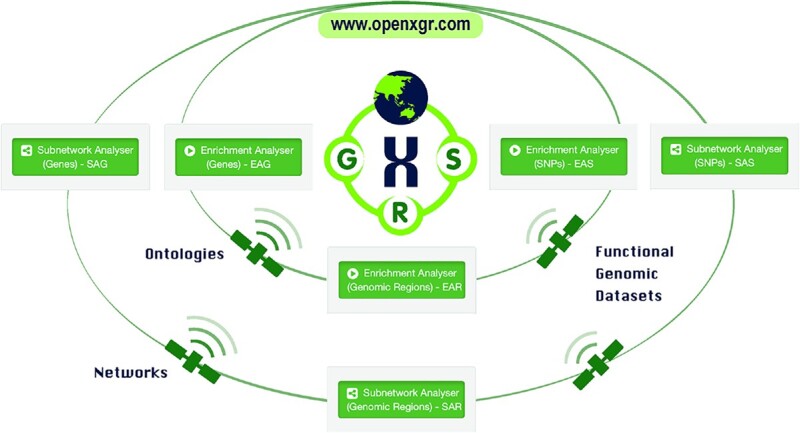
The OpenXGR web server is depicted as our ‘Earth’ in the sense that it comprises six analysers designed to interpret summary-level genomic data related to genes (G), SNPs (S) and genomic regions (R). Similar to satellites that orbit our planet and provide real-time information, OpenXGR leverages knowledgebase on ontologies, networks and functional genomic datasets to enable real-time enrichment and subnetwork analyses.

## INTRODUCTION

Human genomics research produces complex raw genomic data that can be simplified into summary-level data that capture essential information ready for sharing and mining. Without loss of generality, we define genomic summary data as a list of genes, SNPs or genomic regions, along with their summary statistics about the significance level (e.g. *P*-values) (1). Gene-level summary data are often generated from differential expression studies (2), SNP-level summary data from genome-wide association studies (3), and genomic region-level summary data from epigenomic studies (4,5). This simplification of data allows for more straightforward analyses, but how to effectively convert genomic summary data into downstream knowledge discovery remains one of the major challenges in human genomics research.

To address the challenges described above, we have developed e**X**ploring **G**enomic **R**elations or XGR (1) by demonstrating how ontologies enhance genomic summary data interpretation and how to enable insights at the gene subnetwork level. A dozen ontologies have been created to annotate genes regarding functions (6), phenotypes (7,8), diseases (9,10) and other attributes. By integrating a reference gene network that consolidates interaction knowledge (11) with genomic summary data, a subset of the gene network can be identified to best explain the data, thereby gaining insights at the gene subnetwork level. Interpreting non-coding SNPs or genomic regions, however, requires additional use of functional genomic datasets, due to the inherent difficulty in linking them to candidate genes. This difficulty can be resolved by leveraging information from promoter capture Hi-C (PCHi-C) datasets that capture physical interactions with gene promoters (12), quantitative trait loci (QTL) datasets that capture genetic regulation with gene expression (eQTL) (13,14) or protein abundance (pQTL) (15), and datasets about enhancer-gene maps that are constructed using the activity-by-contact (ABC) model (16,17).

**Figure 1.**
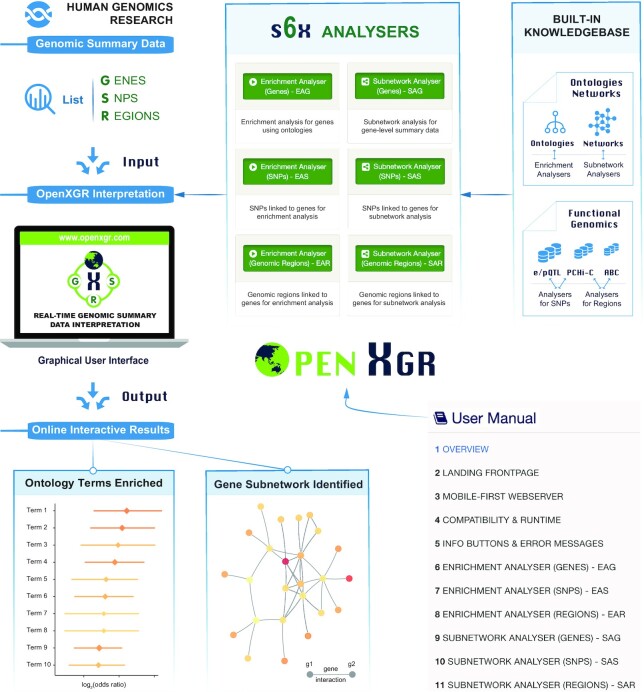
Schematic illustration of how the OpenXGR web server works and what to expect from it. The server at http://www.openxgr.com offers six analysers that are designed to interpret various genomic summary data related to genes (G), SNPs (S) and genomic regions (R). By leveraging built-in knowledgebase on ontologies, networks and functional genomics, these analysers allow almost *real-time* enrichment and subnetwork analyses, enabling identification of ontology enrichments and gene subnetworks. A user manual is made available to provide step-by-step instructions on the use.

Extensively extending our XGR software since its first release (1) and incorporating verified approaches and tools (18–25), in this study, we introduce a newly designed web server ‘OpenXGR’ (Figure 1), which is available at http://www.openxgr.com. Overall, the server is designed to be scalable, efficient and effective, enabling almost *real-time* enrichment and subnetwork analyses for user-input lists of three different entities: genes, SNPs and genomic regions. It is not only limited to the gene- or SNP-centric data types but is also capable of interpreting genomic regions. This generality of capacity for interpreting different entities on the fly is not available elsewhere, thus complementing other popular web servers such as DAVID (26), Enrichr (27) and GREAT (28) that are the most relevant to OpenXGR, and also competitive to standalone tools such as DEPICT (29), MAGMA (30) and jActiveModule (31). OpenXGR achieves this capacity by leveraging increasingly available ontologies, networks, and functional genomic datasets (i.e. PCHi-C, e/pQTL and ABC). Along with a user manual with step-by-step instructions, it offers a user-friendly and all-in-one way to interpret genomic summary data for more integrated and effective knowledge discovery.

In the remaining sections, we will provide an overview of the OpenXGR web server implementation, its six analysers, and the underlying knowledgebase. We will then delve into each analyser that may be of interest to users, with utilities illustrated using practical examples from real-world scenarios, including ageing-related genes (32), gene-level summary data for early human organogenesis (33), SNP-level summary data for chronic inflammatory diseases (34), and genomic region-level summary data for innate immune activation and tolerance (4). Finally, we will conclude with the discussion on the limitations of OpenXGR and the directions for future developments.

## MATERIALS AND METHODS

### Implementation of the OpenXGR web server

The OpenXGR web server (Figure 1) was newly implemented using the Perl real-time web framework ‘Mojolicious’ (https://mojolicious.org) and the widely-used ‘Bootstrap’ (https://getbootstrap.com) to create a mobile-first and responsive design that ensures fast and responsive performance across all major web browsers and mobile devices. All backend computations can be completed within three minutes on the server side to ensure timely delivery of outputs to users. All outputs displayed on the results page are generated using the R package ‘bookdown’ (https://bookdown.org), providing users with a self-contained dynamic HTML file for download and exploration. Additionally, a user manual with step-by-step instructions is made available where needed to facilitate ease of use and provide guidance for users.

### Two types of analysers supported by OpenXGR

The OpenXGR web server offers a range of analysers for conducting enrichment and subnetwork analyses leveraging ontologies and networks. Presently, two types of analysers are supported: one for enrichment analysis designed to identify ontology enrichments, and the other for subnetwork analysis designed to identify gene subnetworks.

Enrichment analysis comprises three analysers that identify enriched ontology terms. These analysers take as input a list of genes, SNPs or genomic regions. One-sided Fisher's exact test is used to calculate *Z*-scores, odds ratio with its 95% confidence interval (CI), and false discovery rate (FDR) for measuring the significance of enrichments. The following are the three analysers supported by OpenXGR:

- *Enrichment analyser for genes (EAG)*, which uses gene-centric ontology annotations to perform enrichment analysis.
- *Enrichment analyser for SNPs (EAS)*, which identifies genes linked from input SNPs (alongside the significance information) and conducts ontology enrichment analysis for the linked genes. Linking SNPs to genes is enabled by genomic proximity or using functional genomic datasets about PCHi-C and e/pQTL.
- *Enrichment analyser for genomic regions (EAR)*, which is similar to *EAS* that first identifies genes linked from input genomic regions using functional genomic datasets about PCHi-C and enhancer-gene maps and then conducts ontology enrichment analysis based on the linked genes.

Subnetwork analysis is performed using three analysers that identify gene subnetworks from input gene-, SNP- or genomic region-level summary data. All subnetwork analysers require the input of the information about the significance level (e.g. *P*-values). The subnetwork identification is done via a heuristic solver for the prize-collecting Steiner tree problem, demonstrated to be competitive to other state-of-the-art algorithms (1,24). The significance (*P*-value) of the identified gene subnetwork can be estimated using a degree-preserving node permutation test to count how often it would be expected by chance. The following are the three subnetwork analysers supported by OpenXGR:

- *Subnetwork analyser for genes (SAG)*, which takes as input gene-level summary data to identify a subset of the gene network in a manner that the resulting subnetwork contains a desired number of highly scored and interconnected genes.
- *Subnetwork analyser for SNPs (SAS)*, which identifies a gene subnetwork from input SNP-level summary data. It first uses genomic proximity, e/pQTL or PCHi-C to link SNPs to genes, and then uses information on the linked genes to identify the gene subnetwork.
- *Subnetwork analyser for genomic regions (SAR)*, which is similar to *SAS* that first identifies genes linked from input genomic regions using PCHi-C datasets or enhancer-gene maps, followed by subnetwork analysis based on the linked genes.

### Leveraging knowledgebase on ontologies, networks and functional genomic datasets

Enrichment analysis in OpenXGR is supported by a variety of ontologies that span a wide range of knowledge contexts, ranging from functions and pathways to regulators, from diseases and phenotypes to drugs, and from protein domains and disorders to hallmarks and evolution. Ontologies currently supported are: (a) *functions*: Gene Ontology (GO) (6) (accessed in April 2023), subdivided into GO Biological Process (GOBP), GO Molecular Function (GOMF), and GO Cellular Component (GOCC); (b) *pathways*: KEGG (35) (105.0 release), REACTOME (36) (version 84 release), pathways from MSIGDB (37) (v2023.1.Hs release), and MitoPathways from MitoCarta (38) (3.0 version); (c) *regulators*: ENRICHR Consensus TFs (27) (accessed in April 2023) and TRRUST (39) (2018.04.16 release); (d) *diseases*: Mondo Disease Ontology (MONDO) (9) (v2023-01-04 release), Disease Ontology (10) (March 2023 release), and Experimental Factor Ontology (EFO) for disease traits (40) (3.52.0 release); (e) *phenotypes*: Human Phenotype Ontology (HPO) (8) (June 2022 release) and Mammalian Phenotype Ontology (MPO) (7) (accessed in April 2023); (f) *drugs*: DGIdb druggable categories (41) (2022-Feb release), target tractability buckets (Bucket) (42) (23.02 release), and ChEMBL drug indications (43) (version 32); (g) *domains & disorders*: SCOP (44), Pfam (45), InterPro (46), and Intrinsically Disordered Proteins Ontology (IDPO) (47) and (h) *hallmarks & evolution*: molecular signature hallmarks from MSigDB (48) and Phylostratigraphy (49).

Subnetwork analysis in OpenXGR leverages the knowledge of functional or pathway interaction networks. Functional interaction networks are sourced from the STRING database (11) (version 11.5), with only the ‘experiments’ and ‘databases’ source codes considered. Functional interactions are classified as having the highest confidence (≥0.9), high confidence (≥0.7), and medium confidence (≥0.4). Pathway interaction networks are sourced from the KEGG database (35) (105.0 release), with all individual pathways being merged into a gene network.

In OpenXGR, linking SNPs to genes is enabled through genomic proximity, PCHi-C or e/pQTL, while linking genomic regions to genes is achieved based on PCHi-C or enhancer-gene maps. Functional genomic datasets currently supported include PCHi-C in immune-, blood- and brain-related cell types (50–53), plasma pQTL (15), blood eQTL from the eQTLGene Consortium (14), eQTL in immune-related cell types and brain-related tissues (13,54,55), and enhancer-gene maps in ENCODE or Roadmap cell types constructed using the ABC model (16,17).

## RESULTS

### Capabilities of enrichment analysers in identifying enriched ontology terms from input genes, SNPs or genomic regions

**Figure 2.**
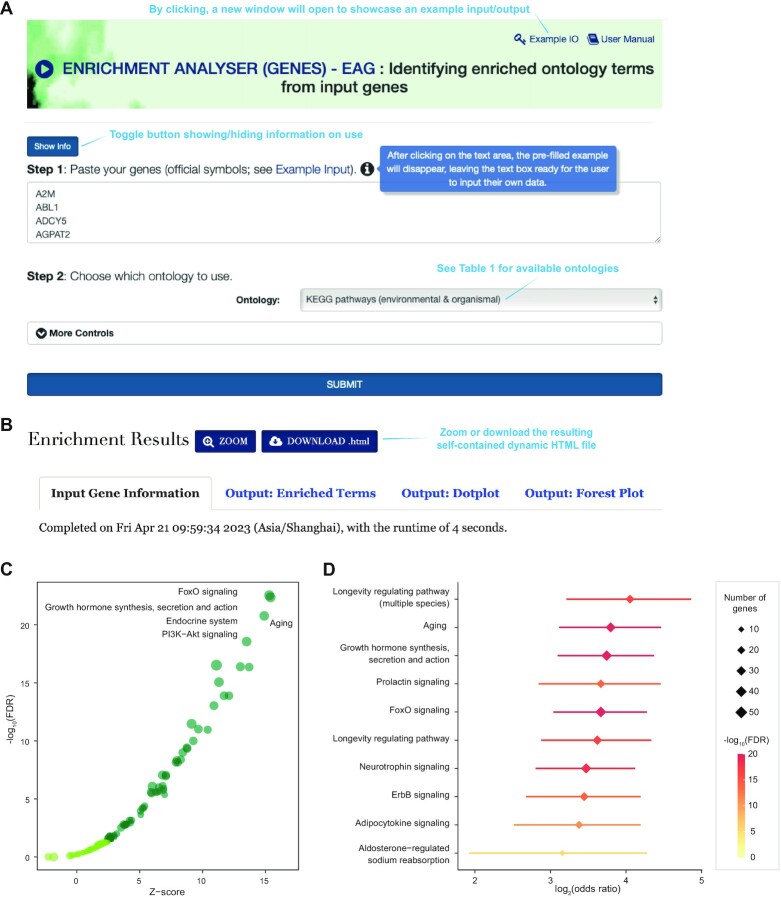
Enrichment analysis for genes using *EAG*. (**A**) User-request interface. The interface takes as input a list of genes and provides available ontologies (listed in Table **1**). **(B)** Enrichment results page. It displays a summary of input data under the ‘*Input Gene Information*’ tab, a table of enriched ontology terms under the ‘*Output: Enriched Terms*’ tab, a dot plot of enrichments under the ‘Output: Dotplot’ tab (see **C**), and a forest plot of enrichments under the ‘*Output: Forest Plot*’ tab (see **D**).

*Enrichment Analyser (Genes)* *– EAG conducts enrichment analysis for genes leveraging* *ontologies*. *EAG* is designed to leverage gene-centric ontology annotations to identify enriched ontology terms from input genes. The tool comprises two major steps, which are outlined in the user-request interface (Figure 2A). The interface takes a list of genes as input, such as ∼300 ageing-related genes as an illustrative example (32). Available ontologies are organised by category (Table 1). Additional parameters can be specified to control the enrichment analysis and results. The interface features a toggle button to show/hide information on the use, including details on input, output and other useful information, as well as a key icon that provides an example input/output showcase. In the results page (Figure 2B), the ‘*Input Gene Information*’ tab lists the input genes and hyperlinks to their corresponding GeneCards pages for additional information and displays the server-side runtime. The ‘*Output: Enriched Terms*’ tab features an interactive table that displays enriched ontology terms, along with their significance information such as Z-scores, FDR, odds ratio and its 95% CI. It also shows member genes that overlap with the input genes. The results are also illustrated in the ‘*Output: Dotplot*’ tab, displaying the top five terms with their respective Z-scores and FDR (Figure 2C), and in the ‘*Output: Forest Plot*’ tab, listing the top enriched terms ordered by odds ratio (Figure 2D). As expected, the most enrichments are ageing-related, such as FoxO signaling, longevity regulating pathways, and ageing. It is worth noting that all enrichment results are embedded into a self-contained dynamic HTML file that can be downloaded and explored interactively in a new browser window. We highly recommend users download this file for subsequent exploration.

**Table 1.** A summary of web browser compatibility, analysers, and built-in knowledgebase (ontologies, networks and genomic datasets) in OpenXGR

Objects	**Characteristics to compare**		
***Web browser compatibility***			
	**MacOS (Big Sur)**	**Windows** **(10** **)**	**Linux (Ubuntu)**
Safari	15.6.1	N/A	N/A
Edge	N/A	108.0.1462.54	N/A
Chrome	108.0.5359.124	108.0.5359.124	108.0.5359.124
Firefox	108.0.1	108.0.1	108.0.1
			
***Analysers***			
	**Type**	**Description**	**Entities**
EAG	Enrichment	Enrichment Analyser (Genes)	Genes
EAS	Enrichment	Enrichment Analyser (SNPs)	SNPs
EAR	Enrichment	Enrichment Analyser (Regions)	Genomic regions
SAG	Subnetwork	Subnetwork Analyser (Genes)	Genes
SAS	Subnetwork	Subnetwork Analyser (SNPs)	SNPs
SAR	Subnetwork	Subnetwork Analyser (Regions)	Genomic regions
			
***Built-in ontologies***			
	**Category**	**Description**	**Used by analysers**
GOBP	Functions	Gene Ontology Biological Process	EAG, EAS, EAR
GOMF	Functions	Gene Ontology Molecular Function	EAG, EAS, EAR
GOCC	Functions	Gene Ontology Cellular Component	EAG, EAS, EAR
KEGG	Pathways	KEGG pathways	EAG, EAS, EAR
REACTOME	Pathways	REACTOME pathways	EAG, EAS, EAR
MSIGDBpath	Pathways	MSIGDB pathways	EAG, EAS, EAR
MITOPATH	Pathways	MitoPathway pathways	EAG, EAS, EAR
CTF	Regulators	ENRICHR Consensus TFs	EAG, EAS, EAR
TRRUST	Regulators	ENRICHR TRRUST TFs	EAG, EAS, EAR
MONDO	Diseases	Mondo Disease Ontology	EAG, EAS, EAR
DO	Diseases	Disease Ontology	EAG, EAS, EAR
EFO	Diseases	Experimental Factor Ontology	EAG, EAS, EAR
HPO	Phenotypes	Human Phenotype Ontology	EAG, EAS, EAR
MPO	Phenotypes	Mammalian Phenotype Ontology	EAG, EAS, EAR
DGIdb	Drugs	DGIdb druggable categories	EAG, EAS, EAR
Bucket	Drugs	Target tractability buckets	EAG, EAS, EAR
ChEMBL	Drugs	ChEMBL drug indications	EAG, EAS, EAR
SCOPsf	Domains & Disorders	SCOP superfamily domains	EAG, EAS, EAR
SCOPfa	Domains & Disorders	SCOP family domains	EAG, EAS, EAR
Pfam	Domains & Disorders	Pfam domains	EAG, EAS, EAR
InterPro	Domains & Disorders	InterPro domains	EAG, EAS, EAR
IDPO	Domains & Disorders	Intrinsically Disordered Proteins Ontology	EAG, EAS, EAR
MSIGDBh	Hallmarks & Evolution	MSIGDB hallmarks	EAG, EAS, EAR
PSG	Hallmarks & Evolution	Phylostratigraphy	EAG, EAS, EAR
		
***Built-in gene networks***			
	**Category**	**Description**	**Used by analysers**
STRING	Functions	Functional interaction networks	SAG, SAS, SAR
KEGG	Pathways	Pathway interaction networks	SAG, SAS, SAR
	
***Built-in functional genomic datasets***			
	**Category**	**Description**	**Used by analysers**
PMID27863249	PCHi-C	Blood-related cell types or states (*n* = 16)	EAS, EAR, SAS, SAR
PMID31501517	PCHi-C	Brain-related tissues (*n* = 3)	EAS, EAR, SAS, SAR
PMID31367015	PCHi-C	Brain-related tissues (*n* = 4)	EAS, EAR, SAS, SAR
PMID25938943	PCHi-C	Blood-related cell types (*n* = 2)	EAS, EAR, SAS, SAR
PMID29875488	pQTL	Plasma eQTL	EAS, SAS
PMID34475573	eQTL	Blood eQTLGen	EAS, SAS
PMID30449622	eQTL	Immune-related cell types (*n* = 15)	EAS, SAS
PMID32913098	eQTL	Brain-related tissues (*n* = 13)	EAS, SAS
PMID33828297	ABC	ENCODE cell types (all combined to be cell type-agnostic)	EAR, SAR
PMID33828297	ABC	Roadmap cell types (all combined to be cell type-agnostic)	EAR, SAR

*Enrichment* *Analyser (* *SNPs) – EAS links SNPs to candidate genes for enrichment analysis*. *EAS* achieves this by linking input SNPs to candidate genes in three steps. The user-request interface requires two pieces of information as input: SNPs and their significance info (p-values). For example, the interface presents an illustrative example of ∼210 SNPs and their reported p-values for chronic inflammatory diseases (34). By default, this analyser considers input SNPs with a *P*-value threshold of <5 × 10^−8^, and additional SNPs in linkage disequilibrium (*R*^2^ ≥0.8) according to the European population, though other populations are also supported (56). Input and additional SNPs are then linked to genes based on genomic proximity, PCHi-C or e/pQTL (see Table 1). The linked genes are scored based on p-values, threshold and *R*^2^ for SNPs, the distance window for genomic proximity, the strength of gene promoters physically interacting with SNP-harbouring genomic regions for PCHi-C datasets, and the significance level defining e/pQTL, as previously described (1,23). Enriched ontology terms are identified based on enrichment analysis of the linked genes. In addition to a dot plot and a forest plot, the output also includes two tabular displays under the ‘*Output: Linked Genes*’ tab. One lists the linked genes and their scores, which range from 1 to 10. The other is an evidence table showing which SNPs are used to define the linked genes based on which datasets.

*Enrichment* *Analyser (* *Genomic Regions) – EAR links genomic regions to candidate genes for enrichment* *analysis*. *EAR* works similarly to *EAS*, but instead of input SNPs, it links input genomic regions to candidate genes and performs enrichment analysis on them. Users specify the genomic coordinates of the input regions, including the chromosome, start, and end positions. The genome build for the input regions is also required, with hg19 used internally as a default and automatically converted if a different build is provided. For example, an input of ∼380 differentially expressed enhancer RNAs (non-coding regions) involved in innate immune activation and tolerance is used as an illustration (4). The linked genes are identified and scored based on genomic proximity, PCHi-C, or enhancer-gene maps (see Table 1). The output includes tabular displays of the linked genes and graphical plots of enriched ontology terms. The linked gene table under the ‘*Output: Linked Genes*’ tab displays information on genes linked from input genomic regions, including scores that quantify the degree to which genes are likely modulated by input genomic regions. The evidence table displays information on which regions are linked to genes based on which evidence. Taken together, *EAR* can handle various genomic regions, such as differentially expressed regions, differentially methylated DNA regions, transcription factor binding sites, and epigenetic marks from epigenomic experiments. It assists in the interpretation by identifying ontology enrichments and candidate genes associated with input genomic regions.

### Capabilities of subnetwork analysers in identifying gene subnetworks from input gene-, SNP- or genomic region-level summary data

**Figure 3.**
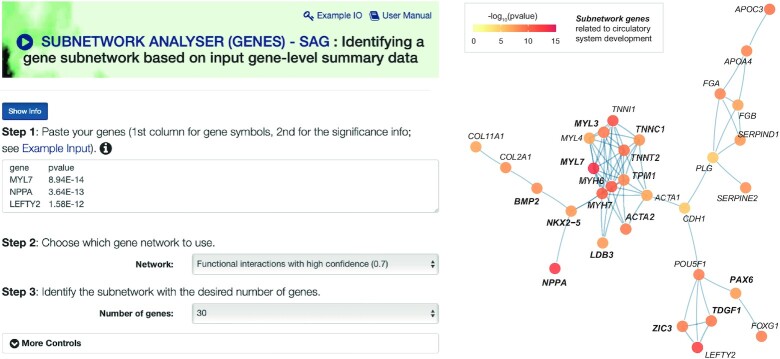
Subnetwork analysis for gene-level summary data using *SAG*. The left panel shows the user-request interface where input gene-level summary data can be provided. The right panel visualises the identified gene subnetwork, with genes/nodes color-coded by their input gene significance information.

*Subnetwork* *Analyser (* *Genes) – SAG performs subnetwork analysis for gene-level summary data leveraging networks*. *SAG* is an analyser designed to exploit knowledge of protein interactions or pathway-derived gene interactions to identify gene subnetworks from input gene-level summary data (Figure 3). A typical example would be a list of differentially expressed genes with their corresponding significance information. An illustrative example provided in the user-input interface is the list of stage-transitive differential genes between Carnegie stages 9 and 10 during early human organogenesis (33). Functional interaction networks are sourced from the STRING database (11), and by default, the high-confidence interactions are used, corresponding to ∼14 800 genes and ∼203 900 interactions. Pathway interaction networks are derived by merging pathways from the KEGG database (35), collectively forming a gene network with ∼6000 genes and ∼59 000 interactions. *SAG* aims to identify a subset of the gene network in such a way that the resulting gene subnetwork (or ‘pathway crosstalk’ if pathway interactions are used) contains most, if not all, of the most significantly and differentially expressed genes in this illustrative example (Figure 3). Interestingly, most of the subnetwork genes are involved in circulatory system development (*ACTA2*, *BMP2*, *LDB3*, *MYH6*, *MYH7*, *MYL3*, *MYL7*, *NKX2-5*, *NPPA*, *PAX6*, *TDGF1*, *TNNC1*, *TNNT2*, *TPM1* and *ZIC3*), amongst which eight genes (*BMP2*, *MYH6*, *MYH7*, *MYL3*, *NKX2-5*, *TNNC1*, *TNNT2* and *TPM1*) are related to cardiac muscle tissue morphogenesis. In other words, the identified subnetwork likely explains primordial development of heart taking place at Carnegie stage 10 (33). Users can specify the desired number of nodes/genes in the resulting subnetwork, and the output is returned via a well-established iterative search procedure (1,24). In summary, *SAG* takes a list of genes along with their significance information, such as differential genes showcased here, and returns a tabular display of the subnetwork genes and a network-like visualisation of the subnetwork (with nodes/genes colour-coded by input gene significance information).

*Subnetwork* *Analyser (* *SNPs) – SAS links SNPs to candidate genes for subnetwork analysis*. *SAS* is designed to perform subnetwork analysis using input SNP-level summary data, with the goal of linking SNPs to genes for subsequent analysis. The first three steps in user-request interface are identical to those in *EAS*. Instead of specifying which ontology to use, users must indicate which gene network to use and provide specifications to control the desired number of the resulting subnetwork genes. Using the same example as in the previous section for *EAS*, under the ‘*Output: Gene Subnetwork*’ tab in the subnetwork results page, the output subnetwork is visualised, with genes/nodes colour-coded by linked gene scores. Interestingly, most of subnetwork genes are involved in C-type lectin receptor signaling (*CARD9*, *CYLD*, *IL10*, *IL12B*, *IL2*, *IRF1*, *NFKB1* and *RHOA*), JAK-STAT signaling (*IL10*, *IL12B*, *IL19*, *IL2*, *IL23R*, *JAK2*, *PDGFB*, *PTPN2* and *SOCS1*), and TNF signaling (*CCL2*, *FOS*, *IRF1*, *NFKB1*, *NOD2* and *TNFRSF1A*). These findings are consistent with the importance of these pathways in inflammation and inflammatory diseases (57–59). In summary, *SAS* is a valuable online tool that links SNPs to genes, enabling the identification of subnetworks that are critical to the understanding of the genetic basis of complex diseases. The resulting gene subnetwork is returned in a tabular display and a network-like visualisation, which facilitates further analysis of candidate genes, particularly enrichment analysis of subnetwork genes to identify enriched pathways.

*Subnetwork* *Analyser (* *Genomic Regions) – SAR links genomic regions to candidate genes for subnetwork analysis*. Similar to *SAS*, this analyser is specially designed for subnetwork analysis using input summary data but at the genomic region level. The first three steps in user-request interface are identical to those in *EAR*. Users need to indicate gene networks to use and specify the desired number of the resulting subnetwork genes. Using real-world summary data on non-coding enhancer RNAs differentially expressed upon innate immune activation and tolerance (4), the output subnetwork is retuned under the tab ‘*Output: Gene Subnetwork*’ in the results page. Enrichment analysis of the resulting subnetwork genes via *EAG* identifies JAK-STAT signaling (*CDKN1A*, *CISH*, *IL10*, *IL10RA*, *IL19*, *IL20*, *IL7*, *IL7R* and *JAK2*) as the most significant pathway (FDR = 2.0 × 10^−6^; odds ratio = 16.0; 95% CI = [6.07, 39.7]), highlighting its crucial role in mediating innate immune activation and tolerance (57).

## DISCUSSION

We have developed OpenXGR to meet the growing demand for efficient and effective interpretation of the ever-increasing volume of summary-level data in genomics. Designed as a versatile and user-friendly web server, it can interpret a wide range of genomic summary data related to three different entities (namely, genes, SNPs and genomic regions). This represents a significant development in human genomics research, as it has the potential to facilitate a more comprehensive understanding of genomic summary data and more effective downstream knowledge discovery.

One of the unique features of OpenXGR is its ability to identify gene subnetworks from input summary data at the gene, SNP and genomic region levels, providing valuable insights into the functional relationships between genes (or linked genes) and aiding in researchers to identify potential pathways or networks that best explain specific biological processes or diseases. Another significant advancement offered by OpenXGR is its use of functional genomic datasets, such as PCHi-C, e/pQTL and enhancer-gene maps, to link non-coding SNPs or genomic regions to candidate genes. This enables interpretation of input SNPs and genomic regions, regardless of their location in the genome, which is often difficult or lacking in existing tools (for interpreting non-coding entities).

However, we recognise that there are limitations to OpenXGR regarding the availability of functional genomic datasets, which currently primarily support blood- and brain-related contexts. Thus, our first aim for future developments is to expand the supporting functional genomic datasets to include a diverse range of cell types, states and tissues. Additionally, enrichment and subnetwork analyses are currently limited to the human genome, so our second aim is to support model organisms, for example, the extension to the mouse genome already on the agenda. This will expand the capacity of OpenXGR in interpreting genomic summary data beyond human. Looking further ahead, we are excited about the opportunity of employing large language models (60) to support genomic summary data interpretation, either in generating ontology annotations and gene networks or in providing outputs in a conversational way similar to ChatGPT. Other future efforts will focus on improving the selection panel of available options (e.g. cell type-specific information on PCHi-C, eQTLs and enhancer-gene maps), supporting enrichment and subnetwork analyses for protein structural domains taken from the dcGO resource (25,61), increasing the user base, and committing to the OpenXGR web server update twice a year. In the long run, OpenXGR will function as an interactive, user-friendly and all-in-one platform that accelerates genomic summary data interpretation by leveraging ontologies, networks, and functional genomics as well.

## DATA AVAILABILITY

The OpenXGR web server is easily accessible at http://www.openxgr.com, where the user manual is also available that provides step-by-step instructions on how to get started (http://www.openxgr.com/OpenXGRbooklet/index.html). The source code is made available on GitHub at https://github.com/hfang-bristol/OpenXGR-site and Figshare at https://doi.org/10.6084/m9.figshare.22679284.v1. For added convenience, OpenXGR can also be accessed through the mirror site at http://www.genomicsummary.com/OpenXGR, along with the user manual at http://www.genomicsummary.com/OpenXGRbooklet/index.html.

## References

[1] Fang H. , Knezevic B., Burnham K.L., Knight J.C. XGR software for enhanced interpretation of genomic summary data, illustrated by application to immunological traits. Genome Med. 2016; 8:129. 27964755 10.1186/s13073-016-0384-y PMC5154134

[2] Stark R. , Grzelak M., Hadfield J. RNA sequencing: the teenage years. Nat. Rev. Genet. 2019; 20:631–656. 31341269 10.1038/s41576-019-0150-2

[3] Tam V. , Patel N., Turcotte M., Bossé Y., Paré G., Meyre D. Benefits and limitations of genome-wide association studies. Nat. Rev. Genet. 2019; 20:467–484. 31068683 10.1038/s41576-019-0127-1

[4] Zhang P. , Amarasinghe H., Whalley J.P., Tay C., Fang H., Migliorini G., Brown A., Allcock A., Scozzafava G., Rath P. et al. Epigenomic analysis reveals a dynamic and context-specific macrophage enhancer landscape associated with innate immune activation and tolerance. Genome Biol. 2022; 23:136. 35751107 10.1186/s13059-022-02702-1 PMC9229144

[5] Kundaje A. , Meuleman W., Ernst J., Bilenky M., Yen A., Heravi-Moussavi A., Kheradpour P., Zhang Z., Wang J., Ziller M.J. et al. Integrative analysis of 111 reference human epigenomes. Nature. 2015; 518:317–330. 25693563 10.1038/nature14248 PMC4530010

[6] Carbon S. , Douglass E., Good B.M., Unni D.R., Harris N.L., Mungall C.J., Basu S., Chisholm R.L., Dodson R.J., Hartline E. et al. The Gene Ontology resource: enriching a GOld mine. Nucleic Acids Res. 2021; 49:D325–D334. 33290552 10.1093/nar/gkaa1113 PMC7779012

[7] Bogue M.A. , Philip V.M., Walton D.O., Grubb S.C., Dunn M.H., Kolishovski G., Emerson J., Mukherjee G., Stearns T., He H. et al. Mouse Phenome Database: a data repository and analysis suite for curated primary mouse phenotype data. Nucleic Acids Res. 2020; 48:D716–D723. 31696236 10.1093/nar/gkz1032 PMC7145612

[8] Köhler S. , Gargano M., Matentzoglu N., Carmody L.C., Lewis-Smith D., Vasilevsky N.A., Danis D., Balagura G., Baynam G., Brower A.M. et al. The human phenotype ontology in 2021. Nucleic Acids Res. 2021; 49:D1207–D1217. 33264411 10.1093/nar/gkaa1043 PMC7778952

[9] Shefchek K.A. , Harris N.L., Gargano M., Matentzoglu N., Unni D., Brush M., Keith D., Conlin T., Vasilevsky N., Zhang X.A. et al. The Monarch Initiative in 2019: an integrative data and analytic platform connecting phenotypes to genotypes across species. Nucleic Acids Res. 2020; 48:D704–D715. 31701156 10.1093/nar/gkz997 PMC7056945

[10] Schriml L.M. , Munro J.B., Schor M., Olley D., McCracken C., Felix V., Baron J.A., Jackson R., Bello S.M., Bearer C. et al. The Human Disease Ontology 2022 update. Nucleic Acids Res. 2022; 50:D1255–D1261. 34755882 10.1093/nar/gkab1063 PMC8728220

[11] Szklarczyk D. , Gable A.L., Nastou K.C., Lyon D., Kirsch R., Pyysalo S., Doncheva N.T., Legeay M., Fang T., Bork P. et al. The STRING database in 2021: customizable protein-protein networks, and functional characterization of user-uploaded gene/measurement sets. Nucleic Acids Res. 2021; 49:D605–D612. 33237311 10.1093/nar/gkaa1074 PMC7779004

[12] Schoenfelder S. , Fraser P. Long-range enhancer–promoter contacts in gene expression control. Nat. Rev. Genet. 2019; 20:437–455. 31086298 10.1038/s41576-019-0128-0

[13] Kerimov N. , Hayhurst J.D., Peikova K., Manning J.R., Walter P., Kolberg L., Samoviča M., Sakthivel M.P., Kuzmin I., Trevanion S.J. et al. A compendium of uniformly processed human gene expression and splicing quantitative trait loci. Nat. Genet. 2021; 53:1290–1299. 34493866 10.1038/s41588-021-00924-w PMC8423625

[14] Võsa U. , Claringbould A., Westra H.-J., Bonder M.J., Deelen P., Zeng B., Kirsten H., Saha A., Kreuzhuber R., Yazar S. et al. Large-scale cis- and trans-eQTL analyses identify thousands of genetic loci and polygenic scores that regulate blood gene expression. Nat. Genet. 2021; 53:1300–1310. 34475573 10.1038/s41588-021-00913-z PMC8432599

[15] Sun B.B. , Maranville J.C., Peters J.E., Stacey D., Staley J.R., Blackshaw J., Burgess S., Jiang T., Paige E., Surendran P. et al. Genomic atlas of the human plasma proteome. Nature. 2018; 558:73–79. 29875488 10.1038/s41586-018-0175-2 PMC6697541

[16] Fulco C.P. , Nasser J., Jones T.R., Munson G., Bergman D.T., Subramanian V., Grossman S.R., Anyoha R., Patwardhan T.A., Nguyen T.H. et al. Activity-by-contact model of enhancer-promoter regulation from thousands of CRISPR perturbations. Nat. Genet. 2019; 51:1664–1669. 31784727 10.1038/s41588-019-0538-0 PMC6886585

[17] Nasser J. , Bergman D.T., Fulco C.P., Guckelberger P., Doughty B.R., Patwardhan T.A., Jones T.R., Nguyen T.H., Ulirsch J.C., Lekschas F. et al. Genome-wide enhancer maps link risk variants to disease genes. Nature. 2021; 593:238–243. 33828297 10.1038/s41586-021-03446-x PMC9153265

[18] Fang H. , Knight J.C. Priority index: database of genetic targets in immune-mediated disease. Nucleic Acids Res. 2022; 50:D1358–D1367. 34751399 10.1093/nar/gkab994 PMC8728240

[19] Bao C. , Wang H., Fang H. Genomic evidence supports the recognition of endometriosis as an inflammatory systemic disease and reveals disease-specific therapeutic potentials of targeting neutrophil degranulation. Front. Immunol. 2022; 13:758440. 35401535 10.3389/fimmu.2022.758440 PMC8983833

[20] Fang H. PiER: web-based facilities tailored for genetic target prioritisation harnessing human disease genetics, functional genomics and protein interactions. Nucleic Acids Res. 2022; 50:W583–W592. 35610036 10.1093/nar/gkac379 PMC9252812

[21] Fang H. , Jiang L. Genetic prioritization, therapeutic repositioning and cross-disease comparisons reveal inflammatory targets tractable for kidney stone disease. Front. Immunol. 2021; 12:687291. 34489936 10.3389/fimmu.2021.687291 PMC8417698

[22] Fang H. , Chen L., Knight J.C. From genome-wide association studies to rational drug target prioritisation in inflammatory arthritis. Lancet Rheumatol. 2020; 2:e50–e62. 10.1016/S2665-9913(19)30134-1 38258277

[23] Fang H. The ULTRA-DD Consortium The ULTRA-DD Consortium De Wolf H. , Knezevic B., Burnham K.L., Osgood J., Sanniti A., Lledó Lara A., Kasela S., De Cesco S. et al. A genetics-led approach defines the drug target landscape of 30 immune-related traits. Nat. Genet. 2019; 51:1082–1091. 31253980 10.1038/s41588-019-0456-1 PMC7124888

[24] Fang H. , Gough J. The ‘dnet’ approach promotes emerging research on cancer patient survival. Genome Med. 2014; 6:64. 25246945 10.1186/s13073-014-0064-8 PMC4160547

[25] Fang H. , Gough J. dcGO: database of domain-centric ontologies on functions, phenotypes, diseases and more. Nucleic Acids Res. 2013; 41:D536–D544. 23161684 10.1093/nar/gks1080 PMC3531119

[26] Sherman B.T. , Hao M., Qiu J., Jiao X., Baseler M.W., Lane H.C., Imamichi T., Chang W. DAVID: a web server for functional enrichment analysis and functional annotation of gene lists (2021 update). Nucleic Acids Res. 2022; 50:W216–W221. 35325185 10.1093/nar/gkac194 PMC9252805

[27] Kuleshov M.V. , Jones M.R., Rouillard A.D., Fernandez N.F., Duan Q., Wang Z., Koplev S., Jenkins S.L., Jagodnik K.M., Lachmann A. et al. Enrichr: a comprehensive gene set enrichment analysis web server 2016 update. Nucleic Acids Res. 2016; 44:W90–W97. 27141961 10.1093/nar/gkw377 PMC4987924

[28] McLean C.Y. , Bristor D., Hiller M., Clarke S.L., Schaar B.T., Lowe C.B., Wenger A.M., Bejerano G. GREAT improves functional interpretation of cis-regulatory regions. Nat. Biotechnol. 2010; 28:495–501. 20436461 10.1038/nbt.1630 PMC4840234

[29] Pers T.H. , Karjalainen J.M., Chan Y., Westra H.J., Wood A.R., Yang J., Lui J.C., Vedantam S., Gustafsson S., Esko T. et al. Biological interpretation of genome-wide association studies using predicted gene functions. Nat. Commun. 2015; 6:5890. 25597830 10.1038/ncomms6890 PMC4420238

[30] de Leeuw C.A. , Mooij J.M., Heskes T., Posthuma D. MAGMA: generalized gene-set analysis of GWAS data. PLoS Comput. Biol. 2015; 11:e1004219. 25885710 10.1371/journal.pcbi.1004219 PMC4401657

[31] Ideker T. , Ozier O., Schwikowski B., Andrew F. Discovering regulatory and signalling circuits in molecular interaction networks. Bioinformatics. 2002; 18:S233–S240. 12169552 10.1093/bioinformatics/18.suppl_1.s233

[32] Tacutu R. , Thornton D., Johnson E., Budovsky A., Barardo D., Craig T., DIana E., Lehmann G., Toren D., Wang J. et al. Human Ageing Genomic Resources: new and updated databases. Nucleic Acids Res. 2018; 46:D1083–D1090. 29121237 10.1093/nar/gkx1042 PMC5753192

[33] Fang H. , Yang Y., Li C., Fu S., Yang Z., Jin G., Wang K., Zhang J., Jin Y. Transcriptome analysis of early organogenesis in human embryos. Dev. Cell. 2010; 19:174–184. 20643359 10.1016/j.devcel.2010.06.014

[34] Ellinghaus D. , Jostins L., Spain S.L., Cortes A., Bethune J., Han B., Park Y.R., Raychaudhuri S., Pouget J.G., Hubenthal M. et al. Analysis of five chronic inflammatory diseases identifies 27 new associations and highlights disease-specific patterns at shared loci. Nat. Genet. 2016; 48:510–518. 26974007 10.1038/ng.3528 PMC4848113

[35] Kanehisa M. , Furumichi M., Sato Y., Kawashima M., Ishiguro-Watanabe M. KEGG for taxonomy-based analysis of pathways and genomes. Nucleic Acids Res. 2023; 51:D587–D592. 36300620 10.1093/nar/gkac963 PMC9825424

[36] Gillespie M. , Jassal B., Stephan R., Milacic M., Rothfels K., Senff-Ribeiro A., Griss J., Sevilla C., Matthews L., Gong C. et al. The reactome pathway knowledgebase 2022. Nucleic Acids Res. 2022; 50:D687–D692. 34788843 10.1093/nar/gkab1028 PMC8689983

[37] Subramanian A. , Tamayo P., Mootha V.K., Mukherjee S., Ebert B.L., Gillette M.A., Paulovich A., Pomeroy S.L., Golub T.R., Lander E.S. et al. Gene set enrichment analysis: a knowledge-based approach for interpreting genome-wide expression profiles. Proc. Natl. Acad. Sci. U.S.A. 2005; 102:15545–15550. 16199517 10.1073/pnas.0506580102 PMC1239896

[38] Rath S. , Sharma R., Gupta R., Ast T., Chan C., Durham T.J., Goodman R.P., Grabarek Z., Haas M.E., Hung W.H.W. et al. MitoCarta3.0: an updated mitochondrial proteome now with sub-organelle localization and pathway annotations. Nucleic Acids Res. 2021; 49:D1541–D1547. 33174596 10.1093/nar/gkaa1011 PMC7778944

[39] Han H. , Cho J.-W., Lee S., Yun A., Kim H., Bae D., Yang S., Kim C.Y., Lee M., Kim E. et al. TRRUST v2: an expanded reference database of human and mouse transcriptional regulatory interactions. Nucleic Acids Res. 2018; 46:D380–D386. 29087512 10.1093/nar/gkx1013 PMC5753191

[40] Sollis E. , Mosaku A., Abid A., Buniello A., Cerezo M., Gil L., Groza T., Güneş O., Hall P., Hayhurst J. et al. The NHGRI-EBI GWAS Catalog: knowledgebase and deposition resource. Nucleic Acids Res. 2023; 51:D977–D985. 36350656 10.1093/nar/gkac1010 PMC9825413

[41] Freshour S.L. , Kiwala S., Cotto K.C., Coffman A.C., McMichael J.F., Song J.J., Griffith M., Griffith O.L., Wagner A.H. Integration of the Drug-Gene Interaction Database (DGIdb 4.0) with open crowdsource efforts. Nucleic Acids Res. 2021; 49:D1144–D1151. 33237278 10.1093/nar/gkaa1084 PMC7778926

[42] Ochoa D. , Hercules A., Carmona M., Suveges D., Baker J., Malangone C., Lopez I., Miranda A., Cruz-Castillo C., Fumis L. et al. The next-generation Open Targets Platform: reimagined, redesigned, rebuilt. Nucleic Acids Res. 2023; 51:D1353–D1359. 36399499 10.1093/nar/gkac1046 PMC9825572

[43] Mendez D. , Gaulton A., Bento A.P., Chambers J., De Veij M., Félix E., Magariños M.P., Mosquera J.F., Mutowo P., Nowotka M. et al. ChEMBL: towards direct deposition of bioassay data. Nucleic Acids Res. 2019; 47:D930–D940. 30398643 10.1093/nar/gky1075 PMC6323927

[44] Murzin A.G. , Brenner S.E., Hubbard T., Chothia C. SCOP: a structural classification of proteins database for the investigation of sequences and structures. J. Mol. Biol. 1995; 247:536–540. 7723011 10.1006/jmbi.1995.0159

[45] Mistry J. , Chuguransky S., Williams L., Qureshi M., Salazar G.A., Sonnhammer E.L.L., Tosatto S.C.E., Paladin L., Raj S., Richardson L.J. et al. Pfam: the protein families database in 2021. Nucleic Acids Res. 2021; 49:D412–D419. 33125078 10.1093/nar/gkaa913 PMC7779014

[46] Blum M. , Chang H.Y., Chuguransky S., Grego T., Kandasaamy S., Mitchell A., Nuka G., Paysan-Lafosse T., Qureshi M., Raj S. et al. The InterPro protein families and domains database: 20 years on. Nucleic Acids Res. 2021; 49:D344–D354. 33156333 10.1093/nar/gkaa977 PMC7778928

[47] Salladini E. , Lazar T., Pancsa R., Chemes B., Pe S., Santos J., Acs V., Farahi N., Dobson L., Chasapi A. et al. DisProt in 2022: improved quality and accessibility of protein intrinsic disorder annotation. Nucleic Acids Res. 2022; 50:D480–D487. 34850135 10.1093/nar/gkab1082 PMC8728214

[48] Liberzon A. , Birger C., Thorvaldsdóttir H., Ghandi M., Mesirov J.P., Tamayo P. The molecular signatures database hallmark gene set collection. Cell Syst. 2015; 1:417–425. 26771021 10.1016/j.cels.2015.12.004 PMC4707969

[49] Trigos A.S. , Pearson R.B., Papenfuss A.T., Goode D.L. Altered interactions between unicellular and multicellular genes drive hallmarks of transformation in a diverse range of solid tumors. Proc. Natl. Acad. Sci. U.S.A. 2017; 114:6406–6411. 28484005 10.1073/pnas.1617743114 PMC5474804

[50] Mifsud B. , Tavares-Cadete F., Young A.N., Sugar R., Schoenfelder S., Ferreira L., Wingett S.W., Andrews S., Grey W., Ewels P.A. et al. Mapping long-range promoter contacts in human cells with high-resolution capture Hi-C. Nat. Genet. 2015; 47:598–606. 25938943 10.1038/ng.3286

[51] Javierre B.M. , Burren O.S., Wilder S.P., Kreuzhuber R., Hill S.M., Sewitz S., Cairns J., Wingett S.W., Várnai C., Thiecke M.J. et al. Lineage-specific genome architecture links enhancers and non-coding disease variants to target gene promoters. Cell. 2016; 167:1369–1384. 27863249 10.1016/j.cell.2016.09.037 PMC5123897

[52] Jung I. , Schmitt A., Diao Y., Lee A.J., Liu T., Yang D., Tan C., Eom J., Chan M., Chee S. et al. A compendium of promoter-centered long-range chromatin interactions in the human genome. Nat. Genet. 2019; 51:1442–1449. 31501517 10.1038/s41588-019-0494-8 PMC6778519

[53] Song M. , Yang X., Ren X., Maliskova L., Li B., Jones I.R., Wang C., Jacob F., Wu K., Traglia M. et al. Mapping cis-regulatory chromatin contacts in neural cells links neuropsychiatric disorder risk variants to target genes. Nat. Genet. 2019; 51:1252–1262. 31367015 10.1038/s41588-019-0472-1 PMC6677164

[54] Schmiedel B.J. , Singh D., Madrigal A., Valdovino-Gonzalez A.G., White B.M., Zapardiel-Gonzalo J., Ha B., Altay G., Greenbaum J.A., McVicker G. et al. Impact of genetic polymorphisms on human immune cell gene expression. Cell. 2018; 175:1701–1715. 30449622 10.1016/j.cell.2018.10.022 PMC6289654

[55] The GTEx Consortium The GTEx Consortium atlas of genetic regulatory effects across human tissues. Science. 2020; 369:1318–1330. 32913098 10.1126/science.aaz1776 PMC7737656

[56] 1000 Genomes Project Consortium An integrated map of genetic variation from 1,092 human genomes. Nature. 2012; 491:56–65. 23128226 10.1038/nature11632 PMC3498066

[57] Banerjee S. , Biehl A., Gadina M., Hasni S., Schwartz D.M. JAK–STAT signaling as a target for inflammatory and autoimmune diseases: current and future prospects. Drugs. 2017; 77:521–546. 28255960 10.1007/s40265-017-0701-9 PMC7102286

[58] del Fresno C. , Iborra S., Saz-Leal P., Martínez-López M., Sancho D. Flexible signaling of Myeloid C-type lectin receptors in immunity and inflammation. Front. Immunol. 2018; 9:804. 29755458 10.3389/fimmu.2018.00804 PMC5932189

[59] van Loo G. , Bertrand M.J.M. Death by TNF: a road to inflammation. Nat. Rev. Immunol. 2022; 23:289–303. 36380021 10.1038/s41577-022-00792-3 PMC9665039

[60] Brown T.B. , Mann B., Ryder N., Subbiah M., Kaplan J., Dhariwal P., Neelakantan A., Shyam P., Sastry G., Askell A. et al. Language models are few-shot learners. Adv. Neural Inf. Process. Syst. 2020; 33:1877–1901.

[61] Bao C. , Lu C., Lin J., Gough J., Fang H. The dcGO domain-centric ontology database in 2023: new website and extended annotations for protein structural domains. J. Mol. Biol. 2023; 435:168093. 37061086 10.1016/j.jmb.2023.168093 PMC7614987

